# Tumour irradiation combined with vascular-targeted photodynamic therapy enhances antitumour effects in pre-clinical prostate cancer

**DOI:** 10.1038/s41416-021-01450-6

**Published:** 2021-06-21

**Authors:** Hanna T. Sjoberg, Yiannis Philippou, Anette L. Magnussen, Iain D. C. Tullis, Esther Bridges, Andrea Chatrian, Joel Lefebvre, Ka Ho Tam, Emma A. Murphy, Jens Rittscher, Dina Preise, Lilach Agemy, Tamar Yechezkel, Sean C. Smart, Paul Kinchesh, Stuart Gilchrist, Danny P. Allen, David A. Scheiblin, Stephen J. Lockett, David A. Wink, Alastair D. Lamb, Ian G. Mills, Adrian Harris, Ruth J. Muschel, Boris Vojnovic, Avigdor Scherz, Freddie C. Hamdy, Richard J. Bryant

**Affiliations:** 1 Nuffield Department of Surgical Sciences, University of Oxford, Oxford, UK; 2 Department of Oncology, University of Oxford, Oxford, UK; 3 Institute of Biomedical Engineering, Department of Engineering Science, University of Oxford, Oxford, UK; 4 Ludwig Institute for Cancer Research, Nuffield Department of Medicine, University of Oxford, Oxford, UK; 5 Target Discovery Institute, NDM Research Building, University of Oxford, Headington, UK; 6 Department of Core Facilities, The Weizmann Institute of Science, Rehovot, Israel; 7 Department of Plant and Environmental Sciences, The Weizmann Institute of Science, Rehovot, Israel; 8 Optical Microscopy and Analysis Laboratory, Frederick National Laboratory for Cancer Research, Leidos Biomedical Research Inc. for the National Cancer Institute, National Institutes of Health, Frederick, MD USA; 9 Cancer and Inflammation Program, Centre for Cancer Research, National Cancer Institute, National Institutes of Health, Frederick, MD USA

**Keywords:** Surgical oncology, Radiotherapy

## Abstract

**Background:**

There is a need to improve the treatment of prostate cancer (PCa) and reduce treatment side effects. Vascular-targeted photodynamic therapy (VTP) is a focal therapy for low-risk low-volume localised PCa, which rapidly disrupts targeted tumour vessels. There is interest in expanding the use of VTP to higher-risk disease. Tumour vasculature is characterised by vessel immaturity, increased permeability, aberrant branching and inefficient flow. FRT alters the tumour microenvironment and promotes transient ‘vascular normalisation’. We hypothesised that multimodality therapy combining fractionated radiotherapy (FRT) and VTP could improve PCa tumour control compared against monotherapy with FRT or VTP.

**Methods:**

We investigated whether sequential delivery of FRT followed by VTP 7 days later improves flank TRAMP-C1 PCa tumour allograft control compared to monotherapy with FRT or VTP.

**Results:**

FRT induced ‘vascular normalisation’ changes in PCa flank tumour allografts, improving vascular function as demonstrated using dynamic contrast-enhanced magnetic resonance imaging. FRT followed by VTP significantly delayed tumour growth in flank PCa allograft pre-clinical models, compared with monotherapy with FRT or VTP, and improved overall survival.

**Conclusion:**

Combining FRT and VTP may be a promising multimodal approach in PCa therapy. This provides proof-of-concept for this multimodality treatment to inform early phase clinical trials.

## Background

There is an unmet clinical need to improve treatment outcomes for clinically significant prostate cancer (PCa) and to reduce treatment side effects. Fractionated radiotherapy (FRT) combined with androgen deprivation therapy (ADT) is a treatment option for PCa, however, a third of cases recur,^1–3^ and subsequent treatment options are limited. Moreover, FRT and ADT have significant side effects. Radical surgery (prostatectomy) is another treatment option for PCa,^4–12^ but this also has significant side effects, emphasising the clinical need for improved treatments.

Vascular-targeted photodynamic therapy (VTP) is a novel minimally invasive focal ablation surgical procedure, achieved by rapid free radical-mediated destruction of tumour vasculature.^13–16^ VTP is effective in focal ablation of low-risk low-volume PCa,^17–19^ and has been investigated as salvage therapy for radio-recurrent PCa.^20,21^ Despite interest in the use of focal ablation therapies such as VTP in higher-risk PCa, to date VTP has not been combined with other treatments as a multimodality therapy approach to high-risk PCa.

VTP involves the intravenous administration of the soluble photosensitising agent WST-11, which conjugates to albumin and is focally activated upon near-infrared illumination in the presence of oxygen. VTP requires a functional tumour vasculature for WST-11 to be efficiently delivered to the target tumour tissue prior to activation by local near-infrared illumination, causing vascular and tumour destruction. Tumour vasculature is typically abnormal and characterised by blood vessel immaturity, increased permeability, increased interstitial pressure, reduced perivascular supporting cell number and function, aberrant vessel branching and inefficient blood flow. These properties impair therapeutic drug delivery and reduce treatment efficacy. FRT alters the tumour microenvironment, and can transiently restore tumour blood vessel function through ‘vascular normalisation’ changes.^22,23^ These ‘vascular normalisation’ changes arise from anti-angiogenesis effects and are defined by pruning of abnormal vessels, reduced vessel tortuosity, improved perivascular supporting cell coverage and function, restoration of the perivascular basement membrane and generation of a pressure gradient between the intravascular and interstitial compartments, leading to more ‘normal’ vessel function and improved perfusion.^24,25^ These ‘vascular normalisation’ changes may influence VTP effectiveness.

Whilst VTP has been investigated in small observation cohort studies of patients with radio-recurrent PCa, the application of VTP during a window of transient ‘vascular normalisation’ changes post-FRT has not been investigated. We investigated combined sequential FRT and VTP as multimodality treatment in a pre-clinical PCa model, to provide proof-of-concept to inform early phase clinical trials. We specifically tested the hypothesis that sequential delivery of FRT followed 7 days later by VTP, during transient post-FRT ‘vascular normalisation’, may improve tumour control compared to FRT or VTP alone.

## Methods

### Cell lines and cell culture

TRAMP-C1 (ATCC^®^ CRL-2730™) and MyC-CaP (ATCC^®^ CRL-3255™) cells purchased from American Type Culture Collection were cultured, as described.^26^ Human umbilical vein endothelial cells (HUVECs) (Lonza, UK) were cultured in EGM-2 medium (Lonza, UK).

### Endothelial growth changes

HUVECs were irradiated with a ^137^Caesium irradiator (IBL 637, CIS Bio International). Cells were exposed to a single 2, 5 or 10 Gy dose of irradiation. Viable (trypan blue-negative) cells were counted over a 7-day time course to assess changes in HUVEC growth. Images of cells in situ, using a white-light microscope, were taken 48 h after RT.

### Endothelial DNA damage assessment

Following 2, 5 or 10 Gy irradiation with a ^137^Caesium irradiator, HUVECs were seeded onto glass slides, and 48 h later stained with 4,6-diamidino-2-phenylindole (DAPI; Invitrogen). Slides were washed with PBS and mounted with Vectashield (Vector Labs). DNA damage was assessed using confocal microscopy (Zeiss 780 inverted confocal microscope) and 405-nm fluorescence excitation.

### Endothelial sprouting assay

Following irradiation of HUVECs with single 2, 5 or 10 Gy doses using a ^137^Caesium irradiator endothelial cell spheroids were generated by the hanging drop method as described.^27^

### In vivo study approval

Animal procedures were performed according to UK Animal law (Scientific Procedures Act 1986) and ARRIVE guidelines, with local ethics and Home Office approval.

### Generation of flank tumour allograft model

Naive 6–8-week old male immunocompetent C57BL/6 and FVB mice (Charles River Laboratories, UK) were housed in groups of six in a pathogen-free facility with 12-h light cycles, in individually ventilated cages on woodchip bedding, with access to water and food ad libitum, at 22 °C (range 21–24 °C) and 50% humidity (range 35–75%), with environmental enrichment and bedding material, and monitored for body weight changes twice weekly.^26^ In total, 2 × 10^6^ TRAMP-C1 (C57BL/6 mice) or 1 × 10^6^ MyC-CaP (FVB mice) cells in PBS and 1:1 high concentration phenol red-free Matrigel^®^ (Corning) were injected into the flank of mice under isofluorane inhalational anaesthesia on a heat mat. Tumours were measured in the home cage pre- and post treatment using digital callipers three times per week (tumour volume = π/6 × length × width × height). When tumours reached 100–120 mm^3^, mice were assigned to treatment groups on a first come, first allocated basis using a randomly generated treatment list (GraphPad Prism 8, USA). All mice were culled by a schedule one procedure at end point in the morning using pentobarbitone injection overdose followed by cervical dislocation according to institutional guidelines.^26^

### Radiotherapy

FRT was delivered as previously described.^26^

### Dynamic contrast-enhanced magnetic resonance imaging

Inhalational anaesthesia was induced and maintained with isofluorane (1–4% in air) in order to maintain a respiration rate of 40–60 breaths per minute, and the temperature was maintained at 35 °C using a homeothermic temperature maintenance system as previously described.^28–30^ Dynamic contrast-enhanced MRI (DCE-MRI) was performed at 4.7 and 7.0 T scanners (Varian, VNMRS console) using 25-mm inner diameter birdcage coils (Rapid Biomedical, Germany) as previously described.^28^ Respiratory-gated 3D gradient-echo scans (echo time = 0.6 ms; repetition time  = 1.15 ms; nominal 5° flip angle) with an isotropic resolution of ∼420 μm and a respiratory rate-dependent frame acquisition time of ∼8–10 s were obtained.^28^ Fifty frames were acquired with a 25 µl bolus infusion of Gadolinium (Gd) solution (Omniscan GE HEALTHCARE) administered using a syringe pump (PHD2000, Harvard Apparatus) over 5 s commencing at the beginning of frame 11. Radiofrequency field inhomogeneities were accounted for using a respiratory-gated implementation of the Actual Flip Angle technique, and baseline T1 was measured using a variable flip angle sequence as reported previously.^28^ In order to analyse tumour perfusion, manual segmentation was initially performed from the average image of the DCE sequence using ITK-SNAP medical image segmentation software.^31^ The MR signal was then converted to Gd concentration, as previously described.^32^ The initial area under the Gd curve (iAUC) was measured by integrating the first 90 s after injection and used as an indicator of perfusion.

### VTP administration

A bespoke optical excitation system was constructed to administer VTP. A small enclosure (Supplementary Fig. 1) contained an animal heating pad, inhalational anaesthesia tubing and physiological monitoring apparatus. Animal imaging, illumination and fibre-coupled excitation and guide beam illumination optics were housed in the enclosure lid. WST-11 VTP excitation was provided by a 755 nm thermoelectrically cooled semiconductor laser diode (LDX Optronix, Missouri, USA, type USA LDX-3210-750), and its output power was controlled by bespoke hardware controlled by a software system and a graphical user interface, which also controlled optical exposure time. A low power (<1 mW) excitation guide beam was provided, generated by a single 590-nm LED (type SMB1N-590-02, Roithner LaserTechnik GmbH, Austria), the output of which was combined with the laser output using a dichromatic reflector (DMLP638R, Thorlabs, UK) prior to launching into the output fibre. A multimode fibre (type M53L02 Ø600 μm, 0.50 NA, Thorlabs, UK) carried both the guide beam and excitation light to the enclosure where it was homogenised and collimated to a slightly diverging beam diameter of 8 mm nominal, delivering a typical excitation intensity of 120 mW/cm^2^. A simple mechanical system and turning prism allowed the excitation and guide beam to be positioned over the area of interest. In addition, the enclosure was fitted with diffuse LEDs operating at 590 nm (Roithner LaserTechnik GmbH, Austria type SMB1N-590-02). The animal in the enclosure could be viewed at all times with the aid of a miniature high dynamic range monochrome camera mounted in the enclosure lid. The wavelength of the guide beam and the illumination (590 nm) was chosen as it was in the nadir of the WST-11 VTP spectral absorption. Similarly, WST-11 injections were performed under yellow light generated by an array of T1¾ 590-nm LEDs (type HLMP-EL3G-VX0DD, Broadcom, USA). Lyophilised WST-11 was a gift from Avigdor Scherz (Weizmann Institute of Science, Israel) and was reconstituted in sterile 5% dextran in water at 2 mg/mL under light protected conditions, and aliquots were stored at −20 °C in the dark and thawed on the day of VTP treatment and sterile filtered through a 0.2-μm disc syringe filter. Mice with 120-mm^3^ tumours received VTP treatment in the morning under isofluorane inhalational anaesthesia with physiological monitoring. Anaesthetised mice received an intravenous infusion of 7 or 9 mg/kg WST-11 via the tail vein followed immediately by 10-min laser excitation of the subcutaneous flank tumour at 120 mW/cm^2^ using a collimating lens. The optical irradiation light field was arranged to cover the entire subcutaneous flank tumour area plus a 1-mm rim. Mice were returned to the home cage following recovery from anaesthesia and underwent post-procedure health monitoring and tumour measurements using digital callipers.

### Immunofluorescence of tissue sections

Freshly excised TRAMP-C1 ttumours were placed into a cryomold filled with optimal cutting temperature compound and quickly frozen in a cold bath containing isopropanol and dry ice. In total, 10-µm frozen tissue sections were cut and placed onto super frost microscope slides (FisherBrand), and fixed with 4% dilution electron microscopy grade paraformaldehyde (Electron Microscopy Sciences) in PBS for 30 min at room temperature in a Coplin jar. Tissue section slides were then washed with PBS in a Coplin jar prior to a combined blocking and permeabilisation step using 3% BSA in 0.3% triton/PBS for 90 minutes at room temperature. Tissue sections were then encircled with a hydrophobic PAP marking pen, and 200 µl of primary antibody (1:200 anti-αSMA antibody, eBioscience, clone 1A4, efluor 570; 1:200 anti-CD31 antibody, Biolegend, clone MEC13.3, Alexa Fluor 488) diluted in blocking solution was added for incubation in a humidified chamber at 4 °C overnight, with this and all subsequent steps under light protection. After removal of primary antibody, a 300 nM DAPI nuclear counterstain in PBS was applied for 30 min incubation prior to washes in PBS, and mounting with coverslips sealed with Wirosil dental silicone. αSMA and CD31 sample images were acquired with a Plan Apo 20 × 0.75 NA objective on a Nikon Ti Widefield Microscope equipped with a Lumencor SpectraX LED light source and an Andor Neo Zyla Camera.

### Digital analysis of tumour vessels

Tumour CD31-positive blood vessel quantification was performed with classical image processing methods. Following image pre-processing, image segmentation was performed to identify tissue areas and CD31-positive blood vessels in an automated fashion. The vessel segmentation was analysed using the Euclidian distance transformation to obtain the local thickness of all segmented objects in the images. The combined binary masks indicating the presence of tissue and CD31-positive blood vessel masks were used to compute the vessel density and the distance from any tissue pixel to the closest vessel.

### Digital annotation and quantification of tumour vessels and αSMA-positive pericytes

α-SMA is often used to identify pericytes,^33^ as these are relatively poorly defined heterogeneous perivascular cell types, without highly specific markers available for their identification.^34^ We used computer-assisted image annotation to identify αSMA-positive pericytes by overlapping the image channels corresponding to αSMA (red) and CD31 (green), and selecting αSMA-positive cells adjacent to, or overlapping, CD31-positive blood vessels. To assist annotation, αSMA-positive cells with a distance of more than 10 pixels, or 3.2 μm, from CD31-positive blood vessels were suppressed. Annotation was performed using the Annotation of Image Data by Assignments (AIDA) web application platform (https://github.com/alanaberdeen/AIDA) developed in-house for the annotation of large microscopy images. Here, relevant regions of interest in the tumour were selected, and the annotators then marked αSMA-positive cells and CD31-positive blood vessels in these regions. Areas in the αSMA channel selected by at least three of four independent observers were accepted as αSMA-positive pericytes. In order to investigate the extent of αSMA-positive pericyte coverage of CD31-positive blood vessels, the fraction of blood vessels in each region with a spatially adjacent αSMA-positive cell was computed. Control untreated samples were compared against FRT-treated samples.

### Statistical analysis

Statistical analysis was performed using GraphPad Prism 8 (GraphPad Software, USA). For in vitro work, ordinary one-way ANOVA tests were performed with Dunnett’s or Tukey’s post hoc adjustment for multiple comparisons. For in vivo tumour growth delay experiments, ordinary one-way ANOVA was performed using Tukey’s test for multiple comparisons following Brown–Forsythe’s test for equality of the means. Tumour growth delay was defined as a significant increase in time (days) for a tumour treated at 100–120 mm^3^ to reach the end-point size of 400 mm^3^ compared against control untreated tumours, a single mouse being considered an experimental unit. All results are mean ±  standard error of the mean. *P* < 0.05 was considered to be a statistically significant difference.

## Results

### VTP monotherapy induces delayed tumour growth in prostate cancer flank tumour allografts

**Fig. 1:**
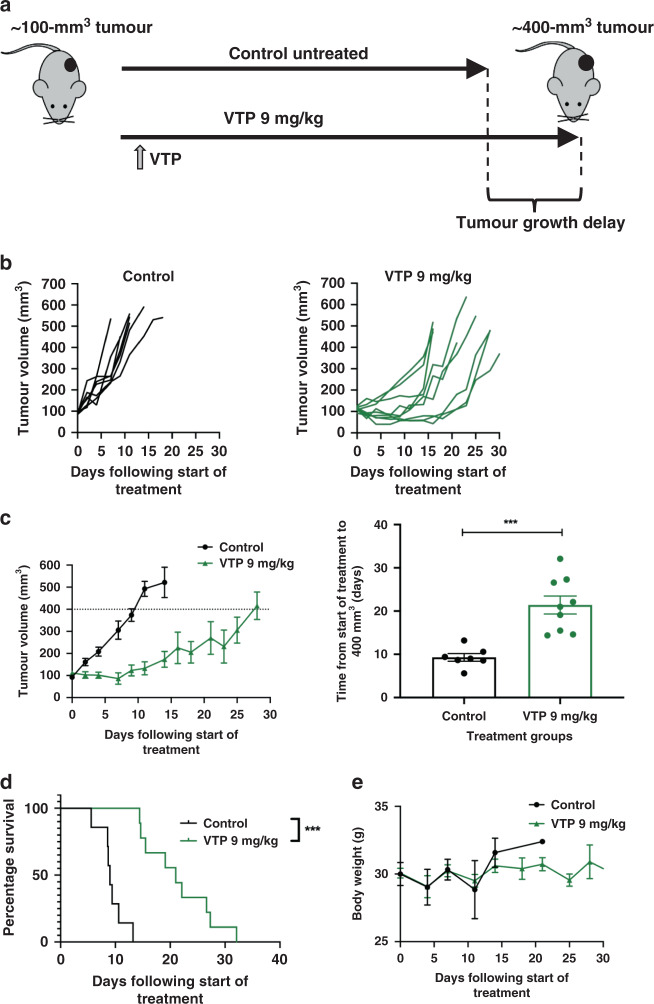
VTP causes tumour growth delay in TRAMP-C1 flank tumour allografts. **a** Outline schematic of treatment of subcutaneous flank TRAMP-C1 tumour allografts with VTP. **b** Growth kinetics of TRAMP-C1 tumours following indicated treatments (*n* = 7 untreated control; *n* = 10 VTP 9 mg/kg WST-11, 120 mW/cm^2^, 600 s). **c** Tumour growth delay to ≥400 mm^3^ analysis of TRAMP-C1 allograft tumours treated with VTP 9 mg/kg WST-11, 120 mW/cm^2^, 600 s. Data are presented as mean tumour volume ± SEM and analysed using ordinary one-way ANOVA with Tukey’s post hoc adjustment for multiple comparisons. *P < 0.05; **P < 0.01; ***P < 0.001, ****P < 0.0001. **d** Treatment of TRAMP-C1 tumour allografts with 9 mg/kg WST-11, 120 mW/cm^2^, for 600 s resulted in enhanced survival to a tumour size of 400 mm^3^ compared with untreated control tumours. **e** Median (range) body weight at start of experiment: untreated control = 21.2 g (20.2–23.5 g); VTP 9 mg/kg = 20.9 g (18.5–23.8 g).

We have previously reported antitumour effects of FRT in a syngeneic immunocompetent TRAMP-C1 PCa flank tumour allograft model.^26^ To similarly investigate antitumour effects of VTP in this model and subsequently combine FRT and VTP as multimodality therapy, we developed an enclosed optical irradiation system to deliver VTP to flank allograft tumours (Supplementary Fig. 1). A bespoke cradle was constructed to accommodate the anaesthetised animal in this optical system. Flank TRAMP-C1 tumour allografts were treated at 100 mm^3^ volume with 9 mg/kg WST-11 at 120 mW/cm^2^ for 600 s, resulting in tumour growth delay to a final tumour size ≥400 mm^3^ compared with untreated control tumours, with no significant welfare implications (Fig. 1).

### Radiotherapy induces ‘vascular normalisation’ changes in prostate cancer flank tumour allografts

To assess the potential for FRT to induce vascular changes in PCa tumours in vivo that might influence VTP treatment, flank TRAMP-C1 tumour allografts were treated with 3 × 5 Gy FRT at 100 mm^3^ and harvested at 7 days, or at ≥400 mm^3^ tumour regrowth end point, following initiation of treatment (Supplementary Fig. 2A). CD31 and αSMA expression was analysed in tissue sections from control untreated and FRT-treated TRAMP-C1 tumours (Supplementary Fig. 2B). Image segmentation analysis revealed a reduced proportion of smaller diameter CD31-positive vessels at 7 days post initiation of FRT versus untreated control tumours (Supplementary Fig. 2C), whilst no difference was observed at ≥400 mm^3^ tumour recurrence post-FRT. The density of CD31-positive tumour vessels (or the number of vessels/cm^2^) was lower for FRT-treated tumours than for untreated control tumours at 7 days post initiation of FRT (Supplementary Fig. 2D). Two-dimensional Euclidian distance transformation analysis of the distance between any tissue image pixel and the closest CD31-positive vessel segment revealed a reduced frequency of short distances between any pixel and the closest CD31-positive vessel at 7 days post initiation of FRT versus untreated control tumours (Supplementary Fig. 2E). This demonstrates that there were longer distances between CD31-positive vessels at 7 days post initiation of FRT compared to control untreated tumours. No difference was observed at ≥400 mm^3^ tumour recurrence post-FRT.

**Fig. 2:**
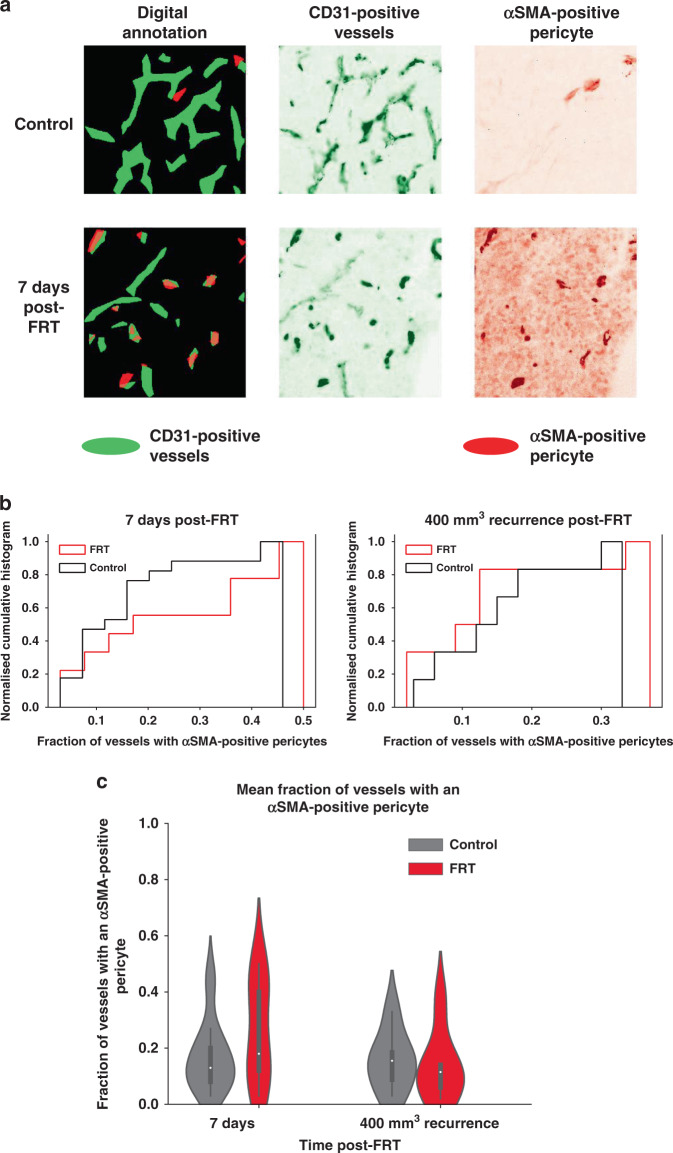
FRT induces ‘vascular normalisation’ changes in flank TRAMP-C1 PCa tumour allografts. **a** Image annotation analysis of immunofluorescence images from control and FRT-treated TRAMP-C1 flank tumours (green—anti-CD31; red— anti-αSMA) revealed a trend **b** towards an increased fraction of vessels with ≥1 adjacent αSMA-positive pericyte at 7 days post-FRT. **c** A trend towards an increased mean fraction of CD31-positive tumour vessels with an adjacent αSMA-positive pericyte was observed 7 days post-FRT compared to control tumours (not statistically significant; overlapping 95% confidence intervals in violin plot).

The association of digitally annotated αSMA-positive pericyte cells with CD31-positive vessels was analysed in TRAMP-C1 flank tumour allografts following FRT (Fig. 2a). An increased fraction of CD31-positive tumour vessels with ≥1 adjacent αSMA-positive pericyte was observed in TRAMP-C1 flank tumour allografts at 7 days post initiation of FRT compared to untreated control tumours (Fig. 2b). This effect was not seen at ≥400-mm^3^ tumour regrowth end-point post-FRT. An increased mean fraction of CD31-positive vessels with an adjacent αSMA-positive pericyte was observed in TRAMP-C1 flank tumour allografts at 7 days post initiation of FRT compared to untreated control tumours (Fig. 2c). No difference was observed between FRT-treated tumours and untreated control tumours at ≥400-mm^3^ tumour regrowth end-point post-RT.

### Radiotherapy improves perfusion in prostate cancer flank tumour allografts

**Fig. 3:**
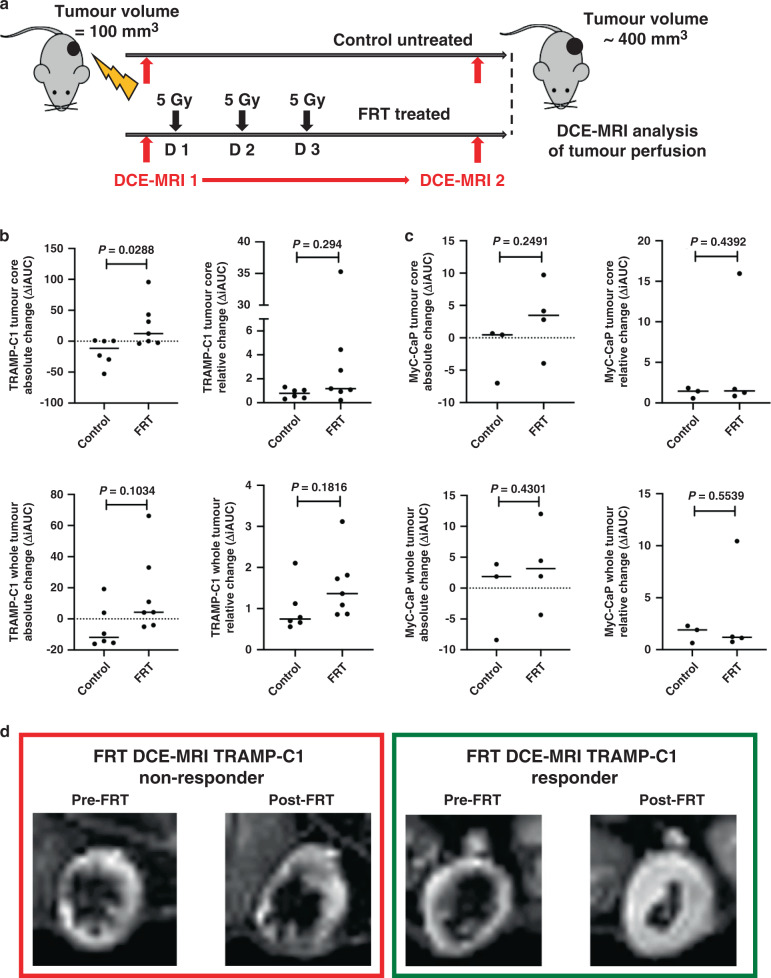
FRT improves vascular perfusion of flank TRAMP-C1 PCa tumour allografts as demonstrated using DCE-MRI. **a** Outline schematic of sequential DCE-MRI imaging of flank TRAMP-C1 tumour allografts immediately pre- and 7 days post-FRT. **b** Analysis of changes in the iAUC in paired image samples pre- and post treatment revealed a significant increase in contrast uptake in the central core of TRAMP-C1 tumours (*P* = 0.0288), and a trend towards an increase in contrast uptake in the entire TRAMP-C1 tumour, following FRT versus control tumours (*P* > 0.05 for all other analyses). **c** Analysis of paired image samples pre- and post-FRT in MyC-CaP tumours revealed a similar trend towards an increase in contrast uptake in both the central core and the entire tumour. **d** An example of a non-responding TRAMP-C1 tumour, and a responding TRAMP-C1 tumour with enhanced vascular perfusion on DCE-MRI, after FRT. Data are presented on a scatter plot with a line representing the median and analysed using a two-tailed unpaired *t* test (*n* = 6 untreated control tumours; *n* = 7 FRT-treated tumours). Median (range) body weight at start of experiment: untreated control tumours = 22.2 g (21.0–24.7 g); FRT-treated tumours = 21.5 g (20.0–25.6 g).

DCE-MRI analysis was performed to investigate whether FRT influences perfusion in TRAMP-C1 flank tumour allografts at 7 days following initiation of FRT (Fig. 3a). FRT enhanced perfusion, as measured by the iAUC at 90 s after Gd injection, 7 days post-FRT in TRAMP-C1 flank tumour allografts (Fig. 3b). Gd contrast-induced enhancement of the fraction of voxels assessed by DCE-MRI was indicative of an improvement of tumour perfusion post-FRT, and this enhanced perfusion occurred predominantly in the tumour core (the central 1/5th of the tumour segmentation by volume) (*P* = 0.0288) (Fig. 3b). Experiments using MyC-CaP tumours in FVB mice as a second tumour model demonstrated a similar trend towards enhanced tumour perfusion at 7 days post-FRT (Fig. 3c).

### Radiotherapy induces in vitro anti-angiogenesis of endothelial cells

**Fig. 4:**
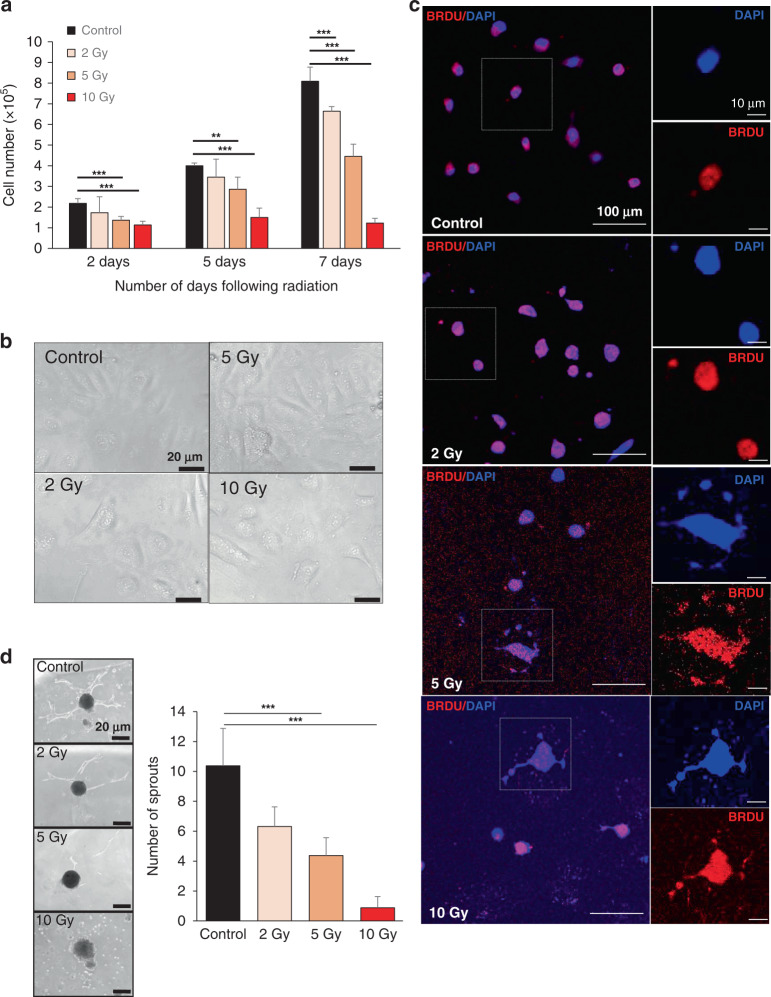
In vitro investigation of effects of irradiation on endothelial cell sprouting and angiogenesis. **a**–**c** A time-course experiment following exposure of human umbilical vein endothelial cells (HUVECs) to 2, 5 or 10 Gy irradiation demonstrated reduced HUVEC cell number, morphological cell changes and increased number of cytoplasmic DNA fragments (DAPI, blue). **d** A ‘hanging drop’ assay demonstrated that endothelial cell sprouting was reduced with increasing irradiation dose at 48 h. Error bars =  standard deviation. **P < 0.01, ***P < 0.0001; one-way ANOVA or unpaired Student’s *t* test comparing two data groups; data representative of *n* = 3 independent experiments.

The in vivo findings in TRAMP-C1 flank tumour allografts 7-days post-FRT demonstrated that CD31-positive tumour vessels were larger in size, fewer in number, further apart, with enhanced αSMA-positive pericyte coverage, and these tumours had enhanced vascular perfusion on DCE-MRI. This indicated that FRT had induced ‘vascular normalisation’ changes by 7-days post-FRT in TRAMP-C1 flank tumours. To investigate the effects of irradiation on endothelial cell sprouting and angiogenesis in vitro, HUVECs were exposed to a single 2, 5 or 10 Gy dose. This demonstrated that an increasing irradiation dose reduced the HUVEC cell number over time following treatment, compared against untreated control cells (Fig. 4a–c). Using an in vitro hanging drop assay, we observed that HUVEC endothelial cell sprouting was reduced with increasing irradiation dose at 48 h post treatment (Fig. 4d).

### Combination therapy using FRT followed by VTP suppressed tumour growth to a greater extent than either treatment alone

**Fig. 5:**
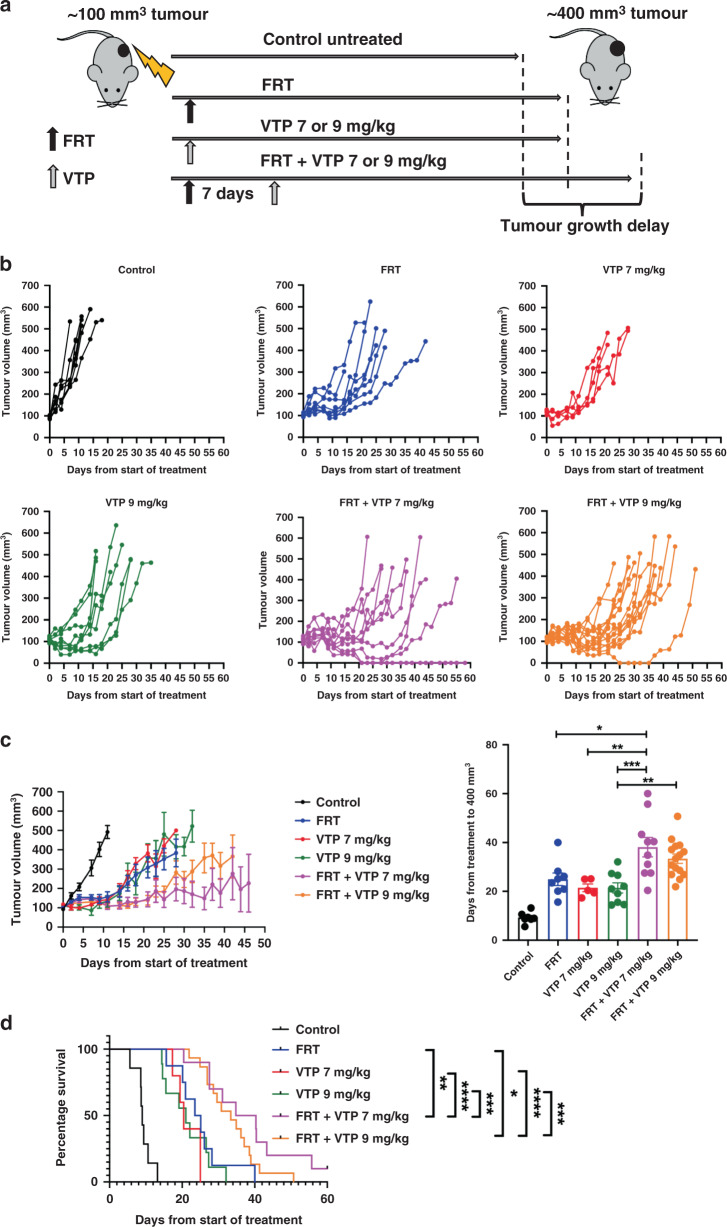
Multimodality therapy with FRT followed at 7 days by VTP causes tumour growth delay in TRAMP-C1 flank tumour allografts. **a** Outline schematic of treatment of tumours with FRT, VTP (7 or 9 mg/kg WST-11), or a sequential combination of FRT followed at 7 days by VTP (7 or 9 mg/kg WST-11). **b** Tumour growth delay analysis of tumours following treatment with FRT, VTP (7 or 9 mg/kg WST-11), or sequential combined FRT and VTP (7 or 9 mg/kg WST-11). **c** Sequential combined FRT and VTP (7 or 9 mg/kg WST-11) significantly delayed tumour growth compared to either FRT or VTP alone. **d** Mice treated with sequential FRT and VTP (7 or 9 mg/kg WST-11) had significantly improved survival to tumour regrowth end point of 400 mm^3^ compared to treatment with either FRT or VTP alone. Numbers per group: control *n* = 7, FRT *n* = 8, VTP 7 mg/kg *n* = 5, VTP 9 mg/kg *n* = 9, FRT and VTP 7 mg/kg *n* = 10, FRT and VTP 9 mg/kg *n* = 15. Median (range) body weight at treatment: control = 21.1 g (20.8–22.1 g), FRT = 21.1 g (19.8–24.3 g), VTP 7 mg/kg = 21.6 g (20.8–23.8 g), VTP 9 mg/kg = 20.9 g (18.5–23.8 g), FRT and VTP 7 mg/kg = 21.9 g (18.9–24.3 g), FRT and VTP 9 mg/kg = 21.3 g (19.6–23.8 g). Data in treatment groups are presented as individual tumour growth kinetics (**b**), grouped tumour growth kinetics (**c**), mean ± SEM growth delay to ≥400 mm^3^ (**c**), and survival to ≥400 mm^3^ using Kaplan–Meier curves (**d**). Data were analysed using ordinary one-way ANOVA with Tukey’s post hoc adjustment for multiple comparisons (**c**), and log-rank (Mantel–Cox) test (**d**). *P < 0.05; **P < 0.01; ***P < 0.001; ****P < 0.0001.

**Fig. 6:**
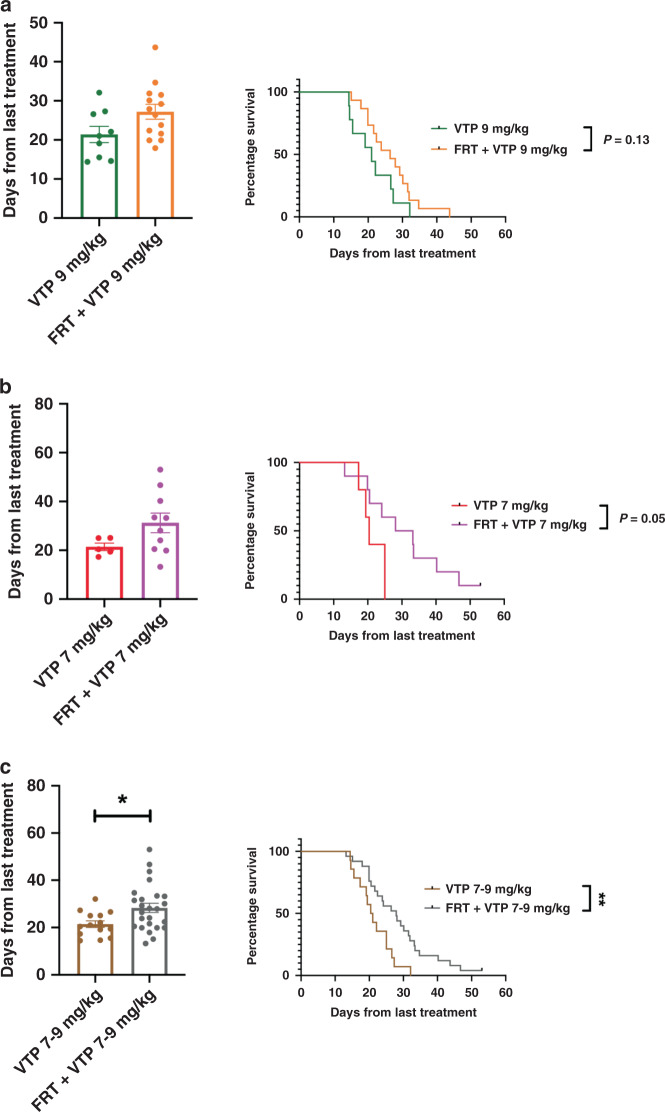
Neoadjuvant FRT improves the efficacy of subsequent VTP delivered 7 days later. Analysis of post-VTP tumour growth delay of TRAMP-C1 flank allograft tumours treated with VTP 7 days post-FRT, compared to VTP alone. **a**, **b** Analysis of time for tumour regrowth from 150 mm^3^ at VTP delivery to the 400 mm^3^ end point revealed a trend towards enhanced tumour growth delay for VTP post-FRT compared to VTP alone for 9 mg/kg WST-11 and 7 mg/kg WST-11. **c** Combined analysis of mice treated with 7–9 mg/kg WST-11 VTP alone versus combined sequential FRT and 7–9 mg/kg WST-11 revealed a significantly enhanced tumour growth delay following administration of VTP if delivered 7 days post-FRT, compared with VTP alone. Data were analysed using a two-tailed unpaired *t* test for the bar chart and log-rank (Mantel–Cox) test for the Kaplan–Meier curves. *P < 0.05; **P < 0.01.

To test the hypothesis that the ‘vascular normalisation’ changes induced by FRT might improve the outcome of VTP-mediated tumour growth control, the effects of sequential treatment of TRAMP-C1 flank allograft tumours with FRT followed by VTP were studied in syngeneic immunocompetent C57BL/6 mice (Fig. 5a). VTP was administered seven days following initiation of FRT to coincide with the timing of the observed FRT-induced ‘vascular normalisation’ changes. Whilst prior studies with VTP as monotherapy had established that 7 mg/kg WST-11 had a lesser efficacy than 9 mg/kg in terms of inducing an antitumour effect, it was tested whether the sequential effects of FRT followed by VTP at seven days might enable a reduction in the WST-11 dose required for tumour growth delay. The effect of FRT followed sequentially at seven days by 7 mg/kg or 9 mg/kg WST-11 VTP was explored (Fig. 5b, c). The sequential combination of FRT followed at 7 days by 9 mg/kg WST-11 VTP delayed tumour growth significantly compared to 9 mg/kg WST-11 VTP alone and led to a trend towards enhanced tumour growth delay compared to FRT alone (Fig. 5b, c). The sequential combination of FRT and 7 mg/kg WST-11 VTP increased significantly tumour growth delay compared to either 7 mg/kg WST-11 VTP alone or FRT alone (Fig. 5b, c). In survival analysis, a sequential combination of FRT and 9 mg/kg WST-11 VTP significantly enhanced survival to a tumour volume ≥400 mm^3^ compared to 9 mg/kg WST-11 VTP alone or FRT alone (Fig. 5d). Similarly, the sequential combination of FRT and 7 mg/kg WST-11 VTP significantly enhanced survival to a tumour volume ≥400 mm^3^ compared to 7 mg/kg WST-11 VTP alone or FRT alone (Fig. 5c). Sequential combination of FRT and 7 mg/kg WST-11 led to the longest survival to ≥400 mm^3^, with one animal being cured (Fig. 5d). 7 mg/kg WST-11 appeared to have greater efficacy than 9 mg/kg WST-11 when combined with FRT. Further analysis of this data demonstrated that the TRAMP-C1 tumour growth delay effects of the VTP component of treatment were enhanced following neoadjuvant FRT, compared against VTP alone. When analysing the time for tumour regrowth from 150 mm^3^ at VTP delivery to ≥400 mm^3^ end point, a trend was observed towards enhanced delay in tumour growth for VTP following neoadjuvant FRT, compared to VTP alone for 9 mg/kg (Fig. 6a) and 7 mg/kg (Fig. 6b) WST-11. A combined analysis of 7–9 mg/kg WST-11 VTP alone versus combined sequential FRT and 7–9 mg/kg WST-11 (Fig. 6c), including all animals in this experiment, showed a significant enhancement in tumour growth delay following VTP delivered 7 days post-FRT, compared with VTP alone.

Finally, we investigated an alternative timing of combination FRT and VTP treatment, to determine if this may be more, or less, effective than FRT followed by VTP at 7 days. We investigated whether 7–9 mg/kg WST-11 VTP delivered during a course of concomitant 3 × 5 Gy FRT led to tumour growth delay, versus the combination with 7–9 mg/kg WST-11 VTP delivered 7 days post-FRT, or versus FRT or VTP alone (Supplementary Fig. 3A). These additional exploratory experiments demonstrated that VTP delivered during a course of concomitant 3 × 5 Gy FRT was no more effective than FRT or VTP monotherapy, and was less effective than 7–9 mg/kg WST-11 VTP delivered 7 days following initial neoadjuvant FRT (Supplementary Fig. 3B).

## Discussion

There is an important clinical need to improve treatment efficacy for PCa, whilst reducing treatment toxicity. Patients with PCa may be offered radical surgery or radical FRT (the latter with neoadjuvant and concomitant ADT for up to three years). However, around a third of patients with high-risk PCa treated with these modalities develop disease recurrence, and these treatments have significant side effects, reducing the quality of life in long-term survivors. There is increasing interest in the role of multimodality therapy for PCa.^35^ To date, PCa is one of few solid organ malignancies where immunotherapy is not part of the standard of care, potentially due to the immunosuppressive tumour microenvironment and/or its low mutational burden.^36,37^ Other treatment combinations beyond the inclusion of immunotherapy may be necessary to improve PCa tumour control and reduce treatment toxicity.

VTP is a minimally invasive focal ablation precision surgical technique, largely evaluated as monotherapy for low-risk low-volume PCa.^17–19,38–40^ VTP has not yet been clinically evaluated in a multimodality therapy approach, or for high-risk PCa. Evidence from several clinical trials in low-risk low-volume disease demonstrates VTP is safe and tolerable.^17–19^ Given that the treatment parameters for VTP have been established for optimal targeted tumour ablation, whilst FRT for PCa is well-established, it could be feasible, based on pre-clinical data, to combine these treatments in early and subsequent late phase clinical trials. Herein, we provide evidence that initial moderate hypofractionated FRT ahead of VTP, with VTP being delivered during a window of time when the irradiated PCa tumour displays ‘vascular normalisation’ changes, leads to enhanced tumour growth delay, with a resultant survival benefit in mice and the possibility of complete tumour cure in some cases. It may be possible, and indeed it is attractive, to combine neoadjuvant FRT with VTP in treating PCa. This may obviate the need for neoadjuvant and/or concomitant ADT, and reduce the dose of conventional FRT administered. This new multimodality therapy approach will require formal clinical evaluation, and in particular will require determination of the optimal dose and fractionation schedule of FRT in patients, combined with the feasibility of performing VTP during the window of ‘vascular normalisation’ changes.

The concept of organ-sparing PCa treatment strategies, directed towards a focal lesion or using a hemi-gland approach to deliver effective tumour control and/or cure with reduced toxicity versus whole-gland treatment, is an increasing focus of PCa research. This approach may have less detrimental effects on quality of life versus the current standard of care of whole-gland therapy.^38,41,42^ VTP has advantages as a focal therapy; VTP has been evaluated in a phase-III randomised clinical trial,^19^ and can anatomise treatment to conform to the malignant lesion along with a margin of normal tissue within the prostate gland, preserving urinary and sexual function. Pre-clinical PCa studies demonstrate that VTP may be successfully combined with ADT,^43^ a 111In-DOTA-AR bombesin antagonist,^44^ and anti-macrophage colony-stimulating factor (anti-CSFR1),^45^, however, VTP has not been investigated in close sequential combination with FRT in pre-clinical or clinical studies. VTP has been demonstrated to be safe and effective in early phase clinical trials in a small number of patients with recurrent PCa following radiotherapy, however, there was a considerable interval of several years between radiotherapy and VTP. The evidence presented herein suggests that sequential combined FRT and VTP within a short interval enhances antitumour control, and warrants evaluation in clinical trials.

The observation that VTP delivered shortly after neoadjuvant FRT increases delay in tumour growth is intriguing and suggests that neoadjuvant FRT delivered in the correct fraction size and dose ahead of subsequent VTP improves the efficacy of this focal therapy. One possible explanation is that transient ‘vascular normalisation’ changes induced by moderate hypofractionated RT transiently increase tumour vessel perfusion, thereby enhancing WST-11 delivery and subsequent tumour ablation by VTP. Experiments with VTP delivered during concomitant FRT showed no benefit from combination, whereas a benefit was seen with VTP delivered 7 days following initiation of FRT, suggesting that the FRT-induced increased tumour perfusion may be important in the efficacy of sequential combination treatment. We observed that FRT enhances tumour perfusion as observed on DCE-MRI 7 days post-FRT, consistent with other studies using similar fractions and doses of FRT.^28^ The dose and fraction size of FRT, along with the window of time in which to look for ‘vascular normalisation’ changes, are key issues as it is known that large irradiation doses rapidly destroy the tumour vasculature, rather than transiently enhancing vascular function and perfusion.^23,46–50^ The results of in vivo xenograft pre-clinical model experiments show that the size and number of irradiation fractions determine whether transient induction of enhanced tumour vascularity occurs. In general: 5–10 Gy fractions cause transient blood flow increase, which returns to normal; 10–15 Gy fractions cause tumour blood flow decrease, which then recovers; and 15–20 Gy fractions cause blood flow to rapidly decrease, and vessels deteriorate.^48,51^ It is important for future clinical translation to consider these aspects of FRT delivery. Hypofractionated RT is increasingly used clinically,^35^ and it would be feasible to use FRT schedules that are likely to induce ‘vascular normalisation’ changes ahead of VTP in a multimodality approach.

We observed that 7 mg/kg WST-11 resulted in a more pronounced reduction in tumour growth following FRT than 9 mg/kg WST-11. One might speculate that near-infrared illumination of 7 mg/kg WST-11 ablates fewer tumour vessels than 9 mg/kg WST-11, leaving sufficient tumour vessels available for immune cells to infiltrate the tumour and elicit an antitumour immune response, thereby enhancing the observed antitumour effects. This intriguing hypothesis warrants investigation in future research.

The concept of targeting the tumour vasculature, rather than the cancer cells, has been explored in multiple pre-clinical and clinical studies. Tumour vessels are often functionally abnormal^52,53^ potentially rendering them susceptible to targeted therapy with molecular agents. In the case of VTP, which requires a functional vasculature, it is possible that prior enhancement of vascular function through ‘vascular normalisation’ changes^22^ improves antitumour effects of subsequent VTP treatment. A variety of vascular targeting agents have been evaluated in pre-clinical cancer research, including inhibitors of angiogenesis and vascular disrupting agents (VDAs). Some VDAs have been investigated in combination with FRT,^54–59^ where the VDA was administered following initial FRT. Combining VDAs with conventional therapies such as FRT may improve treatment outcomes by enhancing antitumour efficacy, with non-overlapping toxicities, and spatial cooperation. VTP may have particular clinical benefit as a VDA compared to drugs such as DMXAA, CA4DP and ZD6126 investigated in other studies. WST-11 used for VTP has minimal toxicity as it is focally activated by near-infrared illumination in the tumour vasculature rather than being active systemically. VTP is therefore a more attractive clinical agent than other VDAs in combination with FRT.

This study has several limitations. Firstly, it has not investigated the immune response to sequential FRT and VTP, and this will be a focus of future research. FRT^26^ and VTP^15,16^ each induce immunological changes within tumours including PCa, and the effects of sequential FRT and VTP on the tumour immune microenvironment will be investigated and reported separately. Secondly, this work was performed on the TRAMP-C1 flank tumour allograft model of PCa in C57BL/6 mice, which is the most commonly used in vivo pre-clinical PCa model. It would be helpful to validate the benefit of combined sequential FRT and VTP in an alternative model of PCa, although the feasibility of combining FRT and VTP in an orthotopic mouse model would be challenging due to concerns over rectal and urethral toxicity of combined treatment. Thirdly, it is an important relative limitation of the reported experiments that they have not incorporated ADT, which is conventionally used in the neoadjuvant clinical setting prior to FRT. ADT could modulate the effect of sequentially combined FRT and VTP, as it is recognised that ADT can affect the tumour vasculature. An extension of the work reported in this manuscript incorporating ADT with combined FRT and VTP will be an important next step to evaluate this multimodality treatment approach. Fourthly, although we demonstrate the growth kinetics of TRAMP-C1 allograft tumours are slower post-VTP following prior FRT, compared to VTP monotherapy, it will be important to investigate the microenvironment of recurrent tumours, as recurrence following combination therapy may promote aggressive disease. Fifthly, we administered a single VTP treatment following FRT, whereas VTP can be repeatedly administered. Finally, given that a functional tumour vasculature and viable endothelial cells after FRT are conventionally considered to be undesirable post-treatment effects as they can promote tumour recurrence and metastasis, the clinical safety of delivering VTP during a window of ‘vascular normalisation’ changes following sublethal FRT requires evaluation.

These findings demonstrate that combined sequential FRT and VTP may be a promising clinical strategy to treat PCa, paving the way for the development of future early phase clinical trials. This approach may also be a useful strategy for other solid organ cancers where improved therapy is desirable.

## Supplementary information

Supplementary Figure Legends

Supplementary Figure 1

Supplementary Figure 2

Supplementary Figure 3

NC3Rs ARRIVE Checklist

## Data Availability

The authors agree to make the data in this paper publically available upon request where possible.

## References

[1] Heidenreich, A., Bastian, P. J., Bellmunt, J., Bolla, M., Joniau, S., van der Kwast, T. et al. EAU guidelines on prostate cancer. Part II: treatment of advanced, relapsing, and castration-resistant prostate cancer. *Eur. Urol.* **65**, 467–479 (2014). 10.1016/j.eururo.2013.11.002 24321502

[2] Grossfeld GD, Li Y, Lubeck DP, Carroll PR (2002). Patterns of failure after primary local therapy for prostate cancer and rationale for secondary therapy. Urology.

[3] Bastian, P. J., Boorjian, S. A., Bossi, A., Briganti, A., Heidenreich, A., Freedland, S. J. et al. High-risk prostate cancer: from definition to contemporary management. *Eur. Urol.* **61**, 1096–1106 (2012). 10.1016/j.eururo.2012.02.031 22386839

[4] Van Poppel, H., Vekemans, K. Da Pozzo, L., Bono, A., Kliment, J., Montironi, R. et al. Radical prostatectomy for locally advanced prostate cancer: Results of a feasibility study (EORTC 30001). *Eur. J. Cancer* **42**, 1062–1067 (2006). 10.1016/j.ejca.2005.11.030 16624554

[5] Carver BS, Bianco FJ, Scardino PT, Eastham JA (2006). Long-term outcome following radical prostatectomy in men with clinical stage T3 prostate cancer. J. Urol..

[6] Freedland, S. J., Partin, A. W., Humphreys, E. B., Mangold, L. A. & Walsh, P. C. Radical prostatectomy for clinical stage T3a disease. *Cancer* **109**, 1273–1278 (2007). 10.1002/cncr.22544 17315165

[7] Hsu CY, Wildhagen MF, Van Poppel H, Bangma CH (2010). Prognostic factors for and outcome of locally advanced prostate cancer after radical prostatectomy. BJU Int..

[8] Xylinas, E., Drouin, S. J., Comperat, E., Vaessen, C., Renard-Penna, R., Misrai, V. et al. Oncological control after radical prostatectomy in men with clinical T3 prostate cancer: a single-centre experience. *BJU Int.* **103**, 1173–1178 (2009). 10.1111/j.1464-410X.2008.08208.x 19040530

[9] Ward JF, Slezak JM, Blute ML, Bergstralh EJ, Zincke H (2005). Radical prostatectomy for clinically advanced (cT3) prostate cancer since the advent of prostate-specific antigen testing: 15-Year outcome. BJU Int..

[10] Loeb S, Smith ND, Roehl KA, Catalona WJ (2007). Intermediate-term potency, continence, and survival outcomes of radical prostatectomy for clinically high-risk or locally advanced prostate cancer. Urology.

[11] Yossepowitch, O., Eggener, S. E., Bianco, F. J., Carver, B. S., Serio, A., Scardino, P. T. et al. Radical prostatectomy for clinically localized, high risk prostate cancer: critical analysis of risk assessment methods. *J. Urol.* **178**, 493–499 (2007). 10.1016/j.juro.2007.03.105 17561152

[12] Zwergel, U., Suttmann, H., Schroeder, T., Siemer, S., Wullich, B., Kamradt, J. et al. Outcome of prostate cancer patients with initial PSA ≥ 20 ng/ml undergoing radical prostatectomy. *Eur. Urol.* **52**, 1058–1066 (2007). 10.1016/j.eururo.2007.03.056 17418938

[13] Madar-Balakirski, N., Tempel-Brami, C., Kalchenko, V., Brenner, O., Varon, D., Scherz, A. et al. Permanent occlusion of feeding arteries and draining veins in solid mouse tumors by vascular targeted photodynamic therapy (VTP) with tookad. *PLoS ONE* **5**, e10282 (2010). 10.1371/journal.pone.0010282 PMC2858664 20421983

[14] Koudinova, N. V., Pinthus, J. H., Brandis, A., Brenner, O., Bendel, P., Ramon, J. et al. Photodynamic therapy with Pd-Bacteriopheophorbide (TOOKAD): successful in vivo treatment of human prostatic small cell carcinoma xenografts. *Int. J. Cancer* **104**, 782–789 (2003). 10.1002/ijc.11002 12640688

[15] Preise, D., Oren, R., Glinert, I., Kalchenko, V., Jung, S., Scherz, A. et al. Systemic antitumor protection by vascular-targeted photodynamic therapy involves cellular and humoral immunity. *Cancer Immunol. Immunother.* **58**, 71–84 (2009). 10.1007/s00262-008-0527-0 PMC11030999 18488222

[16] Preise D, Scherz A, Salomon Y (2011). Antitumor immunity promoted by vascular occluding therapy: lessons from vascular-targeted photodynamic therapy (VTP). Photochem. Photobiol. Sci..

[17] Azzouzi, A. R., Barret, E., Moore, C. M., Villers, A., Allen, C., Scherz, A. et al. TOOKAD® Soluble vascular-targeted photodynamic (VTP) therapy: Determination of optimal treatment conditions and assessment of effects in patients with localised prostate cancer. *BJU Int.* **112**, 766–774 (2013). 10.1111/bju.12265 24028764

[18] Azzouzi, A. R., Barret, E., Bennet, J., Moore, C., Taneja, S., Muir, G. et al. TOOKAD® Soluble focal therapy: pooled analysis of three phase II studies assessing the minimally invasive ablation of localized prostate cancer. *World J. Urol.* **33**, 945–953 (2015). 10.1007/s00345-015-1505-8 PMC4480329 25712310

[19] Azzouzi, A. R., Vincendeau, S., Barret, E., Cicco, A., Kleinclauss, F., van der Poel, H.G. et al. Padeliporfin vascular-targeted photodynamic therapy versus active surveillance in men with low-risk prostate cancer (CLIN1001 PCM301): an open-label, phase 3, randomised controlled trial. *Lancet Oncol.* **18**, 181–191 (2017). 10.1016/S1470-2045(16)30661-1 28007457

[20] Trachtenberg, J., Bogaards, A., Weersink, R. A., Haider, M. A., Evans, A., McCluskey, S. A. et al. Vascular targeted photodynamic therapy with palladium-bacteriopheophorbide photosensitizer for recurrent prostate cancer following definitive radiation therapy: assessment of safety and treatment response. *J. Urol.* **178**, 1974–1979 (2007). 10.1016/j.juro.2007.07.036 17869307

[21] Trachtenberg, J., Weersink, R. A., Davidson, S. R., Haider, M. A., Bogaards, A., Gertner, M. R. et al. Vascular-targeted photodynamic therapy (padoporfin, WST09) for recurrent prostate cancer after failure of external beam radiotherapy: a study of escalating light doses. *BJU Int.* **102**, 556–562 (2008). 10.1111/j.1464-410X.2008.07753.x 18494829

[22] Yoshimura, M., Itasaka, S., Harada, H. & Hiraoka, M. Microenvironment and radiation therapy. *Biomed. Res. Int.* **2013**, 685308 (2013). 10.1155/2013/685308 PMC3591225 23509762

[23] Barker HE, Paget JTE, Khan AA, Harrington KJ (2015). The tumour microenvironment after radiotherapy: mechanisms of resistance and recurrence. Nat. Rev. Cancer.

[24] Goel S, Wong AHK, Jain RK (2012). Vascular normalization as a therapeutic strategy for malignant and nonmalignant disease. Cold Spring Harb. Perspect. Med..

[25] Magnussen, A. L. & Mills, I. G. Vascular normalisation as the stepping stone into tumour microenvironment transformation. *Br. J. Cancer* (2021). 10.1038/s41416-021-01330-z. Online ahead of print. 10.1038/s41416-021-01330-z PMC8329166 33828258

[26] Philippou, Y., Sjoberg, H. T., Murphy, E., Alyacoubi, S., Jones, K. I., Gordon-Weeks, A. N. et al. Impacts of combining anti-PD-L1 immunotherapy and radiotherapy on the tumour immune microenvironment in a murine prostate cancer model. *Br. J. Cancer* **123**, 1089–1100 (2020). 10.1038/s41416-020-0956-x PMC7525450 32641865

[27] Oon, C. E., Bridges, E., Sheldon, H., Sainson, R. C. A., Jubb, A., Turley, H. et al. Role of delta-like 4 in Jagged1-induced tumour angiogenesis and tumour growth. *Oncotarget* **8**, 40115–40131 (2017). 10.18632/oncotarget.16969 PMC5522274 28445154

[28] Kleibeuker, E. A., Fokas, E., Allen, P. D., Kersemans, V., Griffioen, A. W., Beech, J. et al. Low dose angiostatic treatment counteracts radiotherapy-induced tumor perfusion and enhances the anti-tumor effect. *Oncotarget* **7**, 76613–76627 (2016). 10.18632/oncotarget.12814 PMC5363534 27780936

[29] Kersemans, V., Gilchrist, S., Allen, P. D., Beech, J. S., Kinchesh, P., Vojnovic, B. et al. A resistive heating system for homeothermic maintenance in small animals. *Magn. Reson. Imaging* **33**, 847–851 (2015). 10.1016/j.mri.2015.03.011 PMC4462590 25863135

[30] Gilchrist, S., Gomes, A. L., Kinchesh, P., Kersemans, V., Allen, P. D. & Smart, S. C. An MRI-compatible high frequency AC resistive heating system for homeothermic maintenance in small animals. *PLoS ONE* **11**, e0164920 (2016). 10.1371/journal.pone.0164920 PMC5091850 27806062

[31] Yushkevich, P. A., Piven, J., Hazlett, H. C., Smith, R. G., Ho, S., Gee, J. C. et al. User-guided 3D active contour segmentation of anatomical structures: significantly improved efficiency and reliability. *Neuroimage* **31**, 1116–1128 (2006). 10.1016/j.neuroimage.2006.01.015 16545965

[32] Schabel MC, Parker DL (2008). Uncertainty and bias in contrast concentration measurements using spoiled gradient echo pulse sequences. Phys. Med. Biol..

[33] Skalli, O., Pelte, M. F., Peclet, M. C., Gabbiani, G., Gugliotta, P., Bussolati, G. et al. Smooth muscle actin, a differentiation marker of smooth muscle cells, is present in microfilamentous bundles of pericytes. *J. Histochem. Cytochem*. **37**, 315–321 (1989). 10.1177/37.3.2918221 2918221

[34] Yamazaki T, Mukouyama YS (2018). Tissue specific origin, development, and pathological perspectives of pericytes. Front. Cardiovasc. Med..

[35] Philippou Y, Sjoberg H, Lamb AD, Camilleri P, Bryant RJ (2020). Harnessing the potential of multimodal radiotherapy in prostate cancer. Nat. Rev. Urol..

[36] Lawrence, M. S., Stojanov, P., Polak, P., Kryukov, G. V., Cibulskis, K., Sivachenko, A. et al. Mutational heterogeneity in cancer and the search for new cancer-associated genes. *Nature* **499**, 214–218 (2013). 10.1038/nature12213 PMC3919509 23770567

[37] Alexandrov, L. B., Nik-Zainal, S., Wedge, D. C., Aparicio, S. A. J. R., Behjati, S., Biankin, A.V. et al. Signatures of mutational processes in human cancer. *Nature* **500**, 415–241 (2013).

[38] Ahmed HU, Moore C, Emberton M (2009). Minimally-invasive technologies in uro-oncology: The role of cryotherapy, HIFU and photodynamic therapy in whole gland and focal therapy of localised prostate cancer. Surg. Oncol..

[39] Moore CM, Pendse D, Emberton M (2009). Photodynamic therapy for prostate cancer—a review of current status and future promise. Nat. Clin. Pract. Urol..

[40] Moore, C. M., Azzouzi, A. R., Barret, E., Villers, A., Muir, G. H., Barber, N. J. et al. Determination of optimal drug dose and light dose index to achieve minimally invasive focal ablation of localised prostate cancer using WST11-vascular-targeted photodynamic (VTP) therapy. *BJU Int.* **116**, 888–896 (2015). 10.1111/bju.12816 24841929

[41] Valerio, M., Ahmed, H. U., Emberton, M., Lawrentschuk, N., Lazzeri, M., Montironi, R. et al. The role of focal therapy in the management of localised prostate cancer: a systematic review. *Eur. Urol.* **66**, 732–751 (2013). 10.1016/j.eururo.2013.05.048 PMC4179888 23769825

[42] Valerio, M., Cerantola, Y., Eggener, S. E., Lepor, H., Polascik, T. J., Villers, A. et al. New and established technology in focal ablation of the prostate: a systematic review. *Eur. Urol.* **71**, 17–34 (2017). 10.1016/j.eururo.2016.08.044 27595377

[43] Kim, K., Watson, P. A., Lebdai, S., Jebiwott, S., Somma, A. J., La Rosa, S. et al. Androgen deprivation therapy potentiates the efficacy of vascular targeted photodynamic therapy of prostate cancer xenografts. *Clin. Cancer Res.* **24**, 2408–16 (2018). 10.1158/1078-0432.CCR-17-3474 PMC5955858 29463549

[44] Kim, K., Zhang, H. La Rosa, S., Jebiwott, S., Desai, P., Kimm, S. Y. et al. Bombesin antagonist based radiotherapy of prostate cancer combined with WST-11 vascular targeted photodynamic therapy. *Clin. Cancer Res*. **23**, 3343–3351 (2017). 10.1158/1078-0432.CCR-16-2745 PMC5496795 28108545

[45] Lebdai, S., Gigoux, M., Alvim, R., Somma, A., Nagar, K., Azzouzi, A. R. et al. Potentiating vascular-targeted photodynamic therapy through CSF-1R modulation of myeloid cells in a preclinical model of prostate cancer. *Oncoimmunology* **8**, e1581528 (2019). 10.1080/2162402X.2019.1581528 PMC6492957 31069149

[46] Chen, F. H., Chiang, C. S., Wang, C. C., Tsai, C. S., Jung, S. M., Lee, C. C. et al. Radiotherapy decreases vascular density and causes hypoxia with macrophage aggregation in TRAMP-C1 prostate tumors. *Clin. Cancer Res.* **15**, 1721–1729 (2009). 10.1158/1078-0432.CCR-08-1471 PMC2868361 19240176

[47] Park MT, Oh ET, Song MJ, Lee H, Park HJ (2012). Radio-sensitivities and angiogenic signaling pathways of irradiated normal endothelial cells derived from diverse human organs. J. Radiat. Res..

[48] Park HJ, Griffin RJ, Hui S, Levitt SH, Song CW (2012). Radiation-induced vascular damage in tumors: Implications of vascular damage in ablative hypofractionated radiotherapy (SBRT and SRS). Radiat. Res..

[49] Clément-Colmou, K., Potiron, V., Pietri, M., Guillonneau, M., Jouglar, E., Chiavassa, S. et al. Influence of radiotherapy fractionation schedule on the tumor vascular microenvironment in prostate and lung cancer models. *Cancers* **12**, 121 (2020). 10.3390/cancers12010121 PMC7017121 31906502

[50] Supiot, S., Rousseau, C., Dore, M., Chèze-Le-Rest, C., Kandel-Aznar, C., Potiron, V. et al. Reoxygenation during radiotherapy in intermediate-risk prostate cancer. *Radiother. Oncol.* **133**, 16–19 (2019). 10.1016/j.radonc.2018.12.022 30935573

[51] Song, C., Hong, B. J., Bok, S., Lee, C. J., Kim, Y. E., Jeon, S. R. et al. Real-time tumor oxygenation changes after single high-dose radiation therapy in orthotopic and subcutaneous lung cancer in mice: clinical implication for stereotactic ablative radiation therapy schedule optimization. *Int. J. Radiat. Oncol. Biol. Phys.* **95**, 1022–1031 (2016). 10.1016/j.ijrobp.2016.01.064 27130790

[52] Azzi S, Hebda JK, Gavard J (2013). Vascular permeability and drug delivery in cancers. Front. Oncol..

[53] Hanahan D, Weinberg RA (2011). Hallmarks of cancer: the next generation. Cell.

[54] Li L, Rojiani A, Siemann DW (1998). Targeting the tumor vasculature with combretastatin A-4 disodium phosphate: effects on radiation therapy. Int. J. Radiat. Oncol. Biol. Phys..

[55] Siemann DW, Rojiani AM (2002). Enhancement of radiation therapy by the novel vascular targeting agent ZD6126. Int. J. Radiat. Oncol. Biol. Phys..

[56] Wilson WR, Li AE, Cowan DSM, Siim BG (1998). Enhancement of tumor radiation response by the antivascular agent 5,6- dimethylxanthenone-4-acetic acid. Int. J. Radiat. Oncol. Biol. Phys..

[57] Siemann DW, Shi W (2003). Targeting the tumor blood vessel network to enhance the efficacy of radiation therapy. Seminars Radiat. Oncol..

[58] Murata R, Siemann DW, Overgaard J, Horsman MR (2001). Improved tumor response by combining radiation and the vascular-damaging drug 5,6-dimethylxanthenone-4-acetic acid. Radiat. Res..

[59] Landuyt, W., Ahmed, B., Nuyts, S., Theys, J., Op de Beeck, M., Rijnders, A. et al. In vivo antitumor effect of vascular targeting combined with either ionizing radiation or anti-angiogenesis treatment. *Int. J. Radiat. Oncol. Biol. Phys.* **49**, 443–450 (2001). 10.1016/s0360-3016(00)01470-x 11173139

