# Supplementary material for: Tumour irradiation combined with vascular-targeted photodynamic therapy enhances antitumour effects in pre-clinical prostate cancer
Source: Br J Cancer. 2021 Jun 21;125(4):534–46. doi: 10.1038/s41416-021-01450-6 (PMC8367986; doi:10.1038/s41416-021-01450-6)
Supplement: Supplementary file 1 — Supplementary Figure Legends [file 41416_2021_1450_MOESM1_ESM.docx]

**Supplementary Figure 1**

SolidWorks^®^ models used in the design of the VTP optical delivery enclosure. (**A**) View from the front with the front plate transparent to allow internal viewing of the enclosure contents. Homogenised excitation light is delivered via a multimode fibre coupled to a collimator, which delivers a slightly diverging light beam, with spot size adjustment achieved by changing the distance between collimator and prism. A turning prism directs light onto the flank tumour surface. A high dynamic range camera allows viewing of the beam and mouse, which is placed in a cradle. Details of the anaesthesia system are not shown for clarity. (**B**) View of the enclosure with the lid slightly open to reveal the animal illumination, laser interlock switch and details of the adjustable cradle. The whole optical delivery system can be moved laterally to ensure correct illumination of the flank tumour.

**Supplementary Figure 2**

FRT induces ‘vascular normalisation’ changes in flank TRAMP-C1 PCa tumour allografts. (**A**) Outline schematic of treatment of tumours with 3 x 5 Gy FRT ahead of histological analysis of vascular changes. (**B-C**) Image segmentation analysis of immunofluorescence images from untreated control (n=3) and FRT-treated (n=5) TRAMP-C1 flank tumours (green – anti-CD31; red – anti-αSMA) revealed a trend towards a reduced proportion of smaller-diameter blood vessels at 7 days post-initiation of FRT compared to control (not statistically significant as n=4 control and n=4 FRT; overlapping shaded 95% confidence intervals). (**D**) There was a non-significant (*p* = 0.28) trend towards a lower density (number of vessels/cm^2^) of CD31-positive tumor vessels for FRT-treated tumours (n=4) than for control tumours (n=4) at 7 days post-FRT. (**E**) 2D Euclidian distance transformation analysis of the distance between any tissue section pixel and the closest CD31-positive tumour vessel segment in FRT-treated and control TRAMP-C1 tumours. This analysis revealed a trend towards a reduced frequency of short distances between any tissue pixel and the closest CD31-positive tumor vessel at 7 days post-initiation of FRT, indicating that there were longer distances between tumour vessels at this time point (not statistically significant as n=4 control and n=4 FRT; overlapping shaded 95% confidence intervals).

**Supplementary Figure 3**

Multimodality therapy with concomitant FRT and VTP does not increase tumour growth delay in TRAMP-C1 flank tumour allografts compared against monotherapy with FRT or VTP alone, and is less effective in tumour control compared against FRT followed at 7 days by VTP. (**A**) Outline schematic of treatment of tumours with FRT, VTP (7-9 mg/kg WST-11), a sequential combination of FRT followed at 7 days by VTP (7-9 mg/kg WST-11), or concomitant FRT and VTP (7-9 mg/kg WST-11). (**B**) Tumour growth delay analysis of tumours following treatment with FRT, VTP (7-9 mg/kg WST-11), a sequential combination of FRT followed at 7 days by VTP (7-9 mg/kg WST-11), or concomitant FRT and VTP (7-9 mg/kg WST-11). (**C**) A sequential combination of FRT followed at 7 days by VTP (7-9 mg/kg WST-11) significantly delayed tumour growth compared to either FRT or VTP alone. Concomitant FRT and VTP (7-9 mg/kg WST-11) resulted in a similar tumour growth delay to that induced by monotherapy with FRT or VTP (7-9 mg/kg WST-11) alone, and was less effective than sequential FRT followed by VTP (7-9 mg/kg WST-11) at 7 days. (**D**) Mice treated with sequential FRT followed at 7 days by VTP (7-9 mg/kg WST-11) had significantly improved survival to tumour regrowth end-point of 400 mm^3^ compared to treatment with either FRT or VTP alone. However, concomitant FRT and VTP (7-9 mg/kg WST-11) resulted in a similar tumour growth delay to that induced by monotherapy with FRT or VTP (7-9 mg/kg WST-11) alone, and was less effective than sequential FRT followed by VTP (7-9 mg/kg WST-11) at 7 days, therefore this experiment was discontinued with consideration of the 3 R’s for *in vivo* experiments. Numbers per group: control n = 7, FRT n = 9, VTP 7-9 mg/kg n = 15, FRT followed at 7 days by VTP 7-9 mg/kg n = 20, concomitant FRT and VTP 7-9 mg/kg n = 3. Median (range) body weight at treatment: control = 21.2 g (20.8 – 23.5 g), FRT = 21.3 g (19.8 – 24.3 g), VTP 7-9 mg/kg = 21.6 g (18.5 –24.1 g), FRT followed at 7 days by VTP 7-9 mg/kg = 21.9 g (18.9 – 24.3 g), concomitant FRT and VTP 7-9 mg/kg = 21.4 g (20.3 – 21.6 g). Data in treatment groups are presented as individual tumour growth kinetics (**B**), grouped tumour growth kinetics (**C**), mean ± SEM growth delay to ≥400 mm^3^ (**C**), and survival to ≥400 mm^3^ using Kaplan-Meier curves (**D**). Data were analysed using ordinary one-way ANOVA with Tukey’s *post hoc* adjustment for multiple comparisons (C), and Log-Rank (Mantel-Cox) test (D). * *p* < 0.05; ns = not significant, *p*>0.05.
