# Supplementary material for: Tumour irradiation combined with vascular-targeted photodynamic therapy enhances antitumour effects in pre-clinical prostate cancer
Source: Br J Cancer. 2021 Jun 21;125(4):534–46. doi: 10.1038/s41416-021-01450-6 (PMC8367986; doi:10.1038/s41416-021-01450-6)
Supplement: Supplementary file 2 — Supplementary Figure 1 [file 41416_2021_1450_MOESM2_ESM.pptx]

## Slide 1
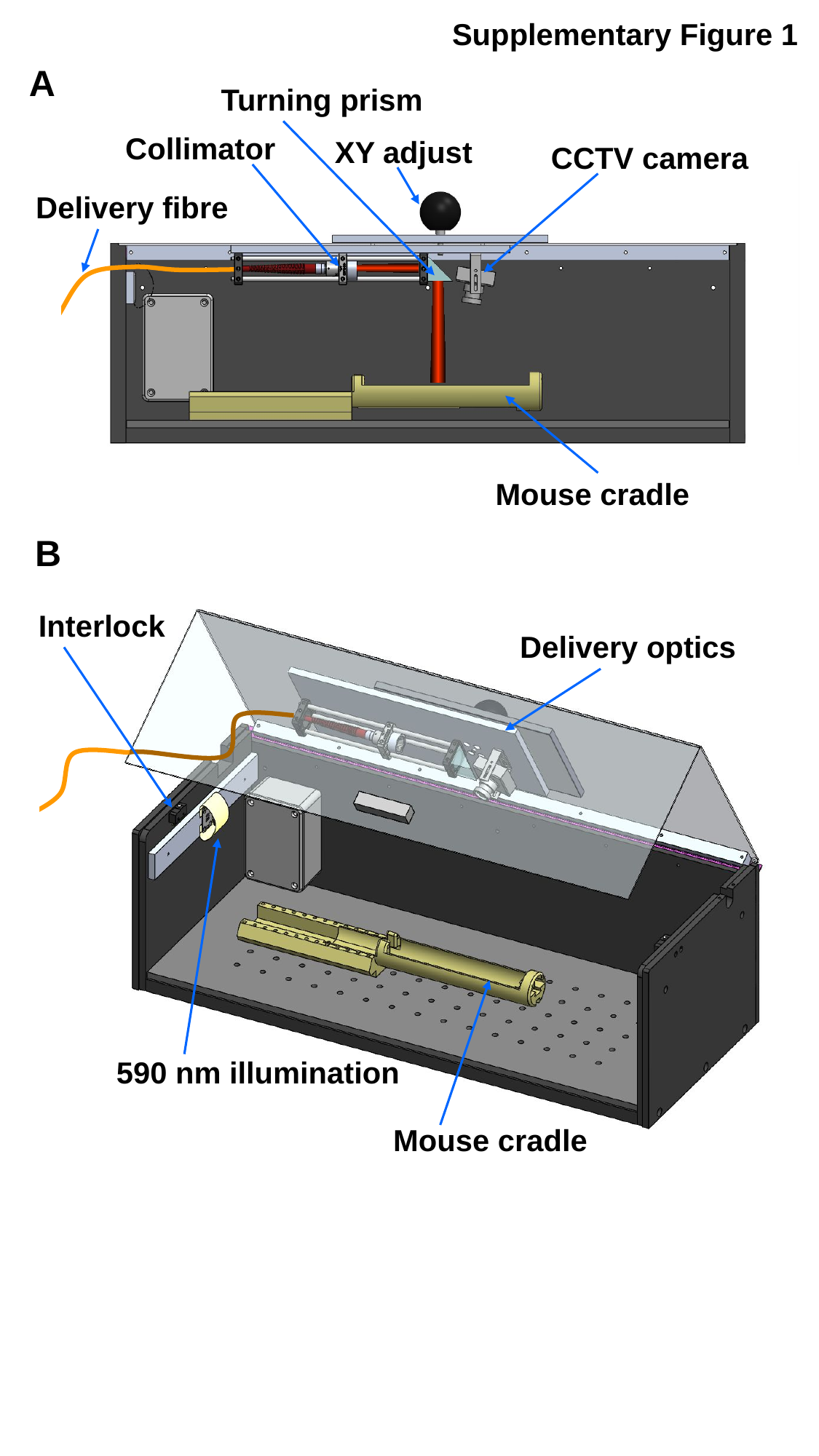

Supplementary Figure 1
A
Turning prism
Collimator
XY adjust
CCTV camera
Delivery fibre
Mouse cradle
B
Interlock
Delivery optics
590 nm illumination
Mouse cradle
