# Supplementary material for: Tumour irradiation combined with vascular-targeted photodynamic therapy enhances antitumour effects in pre-clinical prostate cancer
Source: Br J Cancer. 2021 Jun 21;125(4):534–46. doi: 10.1038/s41416-021-01450-6 (PMC8367986; doi:10.1038/s41416-021-01450-6)
Supplement: Supplementary file 3 — Supplementary Figure 2 [file 41416_2021_1450_MOESM3_ESM.pptx]

## Slide 1
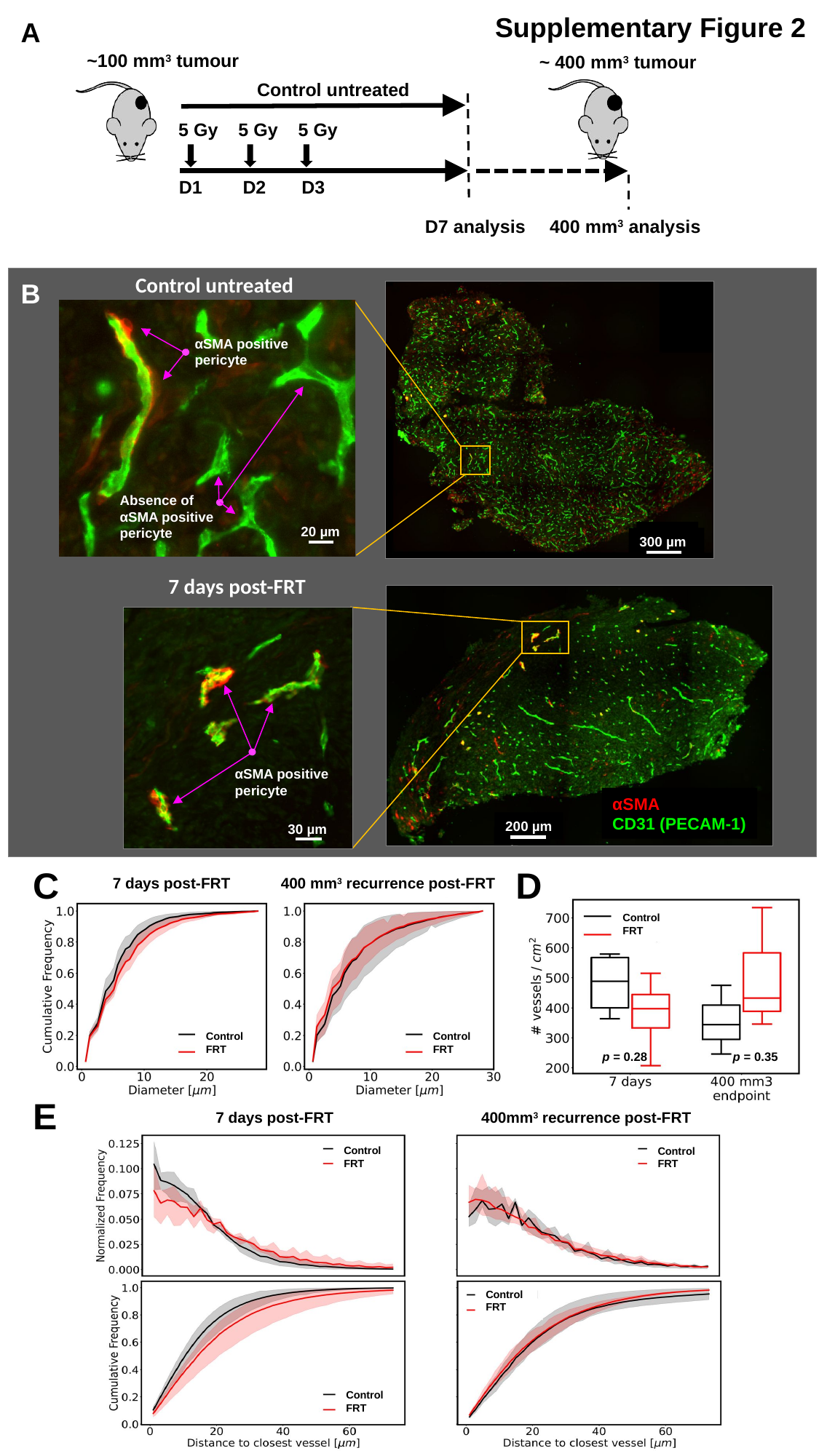

Supplementary Figure 2
A
~100 mm3 tumour
~ 400 mm3 tumour
Control untreated
5 Gy 5 Gy 5 Gy
D1 D2 D3
D7 analysis
400 mm3 analysis
Control untreated
B
B
αSMA positive pericyte
Absence of αSMA positive pericyte
20 µm
300 µm
300 µm
7 days post-FRT
C
αSMA positive pericyte
30 µm
aSMA
PECAM-1 [CD31]
αSMA
CD31 (PECAM-1)
200 µm
200 µm
C
D
7 days post-FRT
400 mm3 recurrence post-FRT
Control
FRT
Control
FRT
Control
FRT
p = 0.28
p = 0.35
E
7 days post-FRT
400mm3 recurrence post-FRT
Control
FRT
Control
FRT
Control
FRT
Control
FRT
Control
FRT
