# Supplementary material for: Tumour irradiation combined with vascular-targeted photodynamic therapy enhances antitumour effects in pre-clinical prostate cancer
Source: Br J Cancer. 2021 Jun 21;125(4):534–46. doi: 10.1038/s41416-021-01450-6 (PMC8367986; doi:10.1038/s41416-021-01450-6)
Supplement: Supplementary file 4 — Supplementary Figure 3 [file 41416_2021_1450_MOESM4_ESM.pptx]

## Slide 1
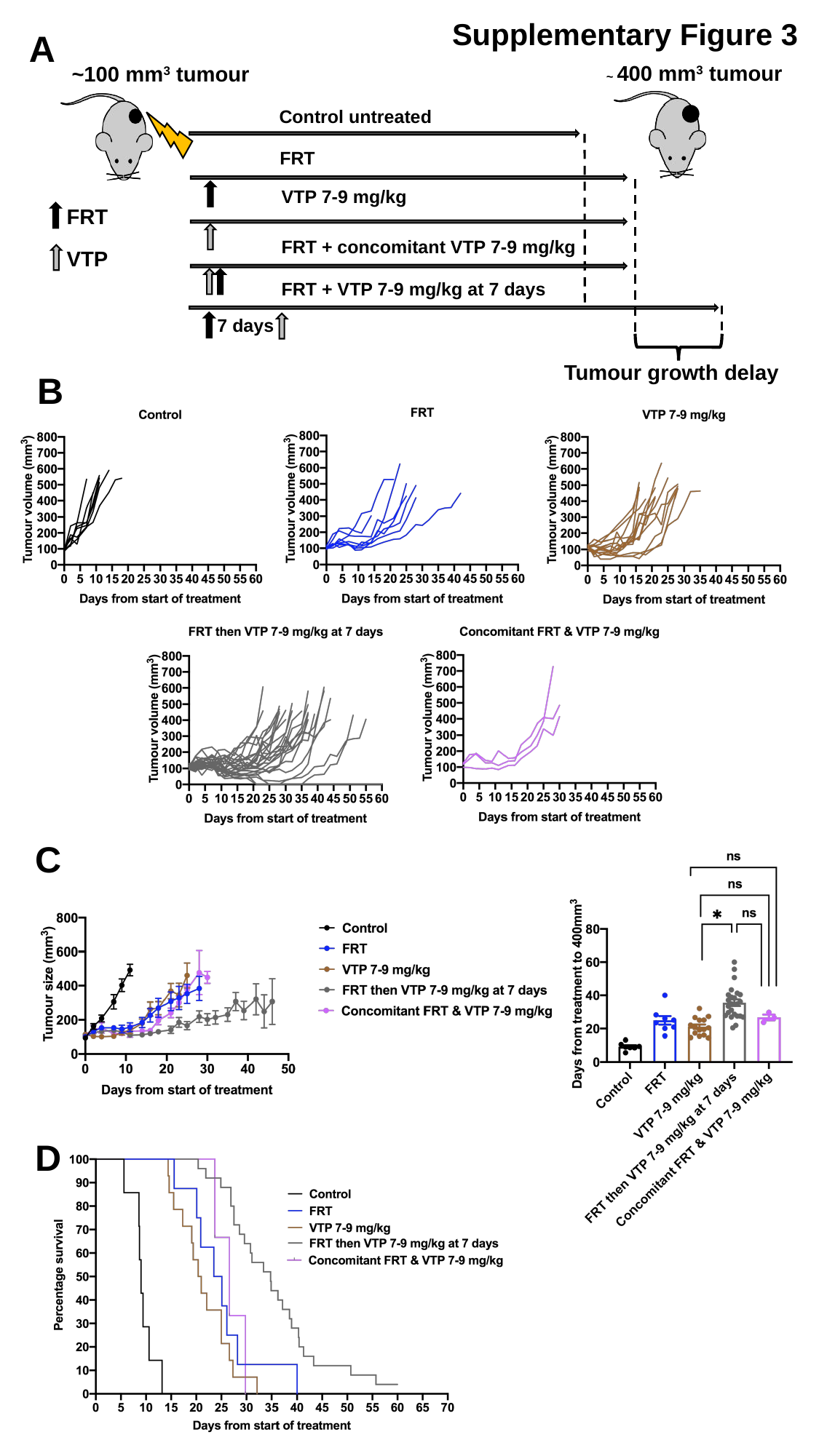

Supplementary Figure 3
A
~ 400 mm3 tumour
~100 mm3 tumour
Control untreated
FRT
VTP 7-9 mg/kg
FRT
FRT + concomitant VTP 7-9 mg/kg
VTP
FRT + VTP 7-9 mg/kg at 7 days
7 days
Tumour growth delay
B
C
D
